# Adipocytes promote metastasis of breast cancer by attenuating the FOXO1 effects and regulating copper homeostasis

**DOI:** 10.1186/s12935-024-03433-y

**Published:** 2024-08-12

**Authors:** Xiu Chen, Heda Zhang, Zheng Fang, Dandan Wang, Yuxin Song, Qian Zhang, Junchen Hou, Sujin Yang, Di Xu, Yinjiao Fei, Wei Zhang, Jian Zhang, Jinhai Tang, Lei Li

**Affiliations:** 1 Department of General Surgery, The First Affiliated Hospital with Nanjing Medical University, Nanjing, 210029 Jiangsu China; 2 Department of General Surgery, The First Affiliated Hospital of Anhui Medical University, Hefei, 230022 Anhui China

**Keywords:** Breast cancer, Adipocyte, FOXO1, Metastasis, Copper homeostasis

## Abstract

**Background:**

Obesity and the forkhead box O1(FOXO1) affect the survival of breast cancer patients, but the underlying mechanism remains unclear. We aimed to investigate the role of FOXO1 in obesity-associated-breast cancer.

**Methods:**

We screened 383 breast disease patients from the first affiliated hospital with Nanjing Medical University in 2020. We performed wound healing, transwell, matrigel assays to assess the metastatic ability of cancer cells. We adopted mRNAs sequencing to select the differentially expressed transcripts in breast cancer. We applied immunohistochemistry, western blot, tissue microarrays to assess the level of FOXO1 and epithelial-mesenchymal transition (EMT) pathways. We conducted bioinformatic analysis to investigate interactions between FOXO1 and miR-135b. We used fluorescence in situ hybridization, RT-qPCR to confirm the characteristics of circCNIH4. We conducted luciferase reporter assay, rescue experiments to investigate interactions between circCNIH4 and miR-135b.

**Results:**

Obesity was positively correlated with the incidence and progression of breast cancer. Adipocytes enhanced the migration of breast cancer and attenuated the effects of FOXO1. MiR-135b was a binding gene of FOXO1 and was regulated by circCNIH4. CircCNIH4 exhibited antitumor activity in vitro and in vivo.

**Conclusion:**

Adipocytes might accelerate the progression of breast cancer by modulating FOXO1/miR-135b/ circCNIH4 /EMT axis and regulating copper homeostasis.

**Supplementary Information:**

The online version contains supplementary material available at 10.1186/s12935-024-03433-y.

## Background

About half of adults were enduring weight problem in Chinese population [1]. In statistic surveys, the overall prevalence of obesity was twice in 2018(8·1%, 95% CI 7·6–8·7) than in 2004(3·1%, 95% CI 2·5–3·7) [2]. Besides, those obese women who were postmenopausal had a nearly 30% higher risk to develop breast cancer [3]. In our study, the body mass index(BMI) was associated with the occurrence of breast cancer which containing approximately 49% obese or overweight patients. This results was in consist with previous literatures. It was known that several solid tumors had close anatomical proximity to adipose tissues, which could provide a hospitable environment for tumor growth [4]. In breast cancer, for instance, obesity was associated with the poorer overall survival and an increased risk of mortality, irrespective of menopausal status [5]. The underlying mechanisms in the development of breast cancer with obesity were mainly as follows: (a) hypoxia related secretion of cytokines and leptins in obese adipose tissue sustained a chronic inflammatory surroundings by expanding proinflammatory immune cells and preadipocyte numbers [6]. (b) high level of insulin and IFG-1 released by the interaction between adipocytes and invading cancer cells induced breast cancer growth [7, 8]. (c) aromatase upregulation in breast stromal fibroblasts and adipocytes increased estrogen production in cancer cells, leading to cancer developments [9]. In our study, we cocultured adipocytes with breast cancer cells and detected that adipocytes accelerated the invasion and metastasis of breast cancer. Therefore, it is indispensable to consider obesity as one of the crucial elements in the treatment of breast cancer.

The epidemiological associations suggested the need for a deeper understanding of the underlying mechanisms linking obesity and breast cancer. So, we co-cultured adipocytes and breast cancer cells and analyzed the changes of mRNAs in breast cancer cells. Results showed that the differentially expressed mRNAs mainly functioned in active transmembrane transporter activities and transcription factor binding pathways. The conserved transcription factor Forkhead box protein O1 (FoxO1) was reported to ameliorate lipid metabolism disorders in obese population [10]. It was tightly regulated by modifications on its mRNA or protein and responded to environmental nutrient signals. However, rare studies had explored the relationship between FOXO1 and obesity in breast cancer patients. So we focused on the effects of FOXO1 in breast cancer after co-cultured with adipocytes.

In our study, adipocytes-induced reduction of FOXO1 mainly regulated the migration and invasion of breast cancer. In other words, the most remarkable regulations in breast cancer brought by FOXO1 was the changes in migration capability, along with the alterations in biomarker proteins of the epithelial-mesenchymal transition. The epithelial-mesenchymal transition (EMT) is a crucial program that drives invasion and metastasis in malignant tumors. The tumor microenvironment appeared to be one of the contributors in causing EMT [11]. In breast cancer, previous researchers have discovered that adipocytes as a predominate essence of tumor microenvironment had potential to induce EMT by various pathways [12, 13]. In our study, we fortunately strengthened the concepts that the FOXO1 served its suppressive role in metastasis of adipocytes-associated breast cancer through EMT pathways.

Bioinformation analysis with previous researches have established FOXO1 as a downstream target of miR-135b in multiple cancer types. After preliminary experiments, miR-135b showed evident relationship with FOXO1 in breast cancer development. The functional experiments then confirmed their correlation. These findings suggested that FOXO1-miR-135b might be important molecular targets for understanding and treating obesity-related cancers.

In several studies, circular RNAs were proven to participate in the mentioned processes of breast cancer development [14–16]. In obese patients, altered expressions of circRNAs was one of the most prominent variations [17, 18]. However, the unique mechanisms of circRNAs in the mutual actions between obesity and breast cancer remained unclear. In this study, we aimed to examine the role of circRNAs in obesity-related breast cancer.

Circular RNAs (circRNAs) are a class of non-coding RNAs that are formed through a back-splicing mechanism of precursor messenger RNAs (pre-mRNAs) of numerous genes. CircRNAs have been shown to modulate gene expression by functioning as miRNA sponges or by binding to RNA-binding proteins (RBPs) to regulate transcription and translation [19–21]. As such, circRNAs have been implicated in biologic process of various human diseases and cancers [22], including tumorigenesis, apoptosis, metastasis [23]. The most common mechanism of circRNAs to function in tumorigenesis and tumor progression was to act as a miRNA sponge. Simultaneously, miRNAs were found to join in the interaction between cancer cells and obesity. Bioinformatics analysis was the most popular instrument to research the relationship between circRNAs and miR-135b-5p, then we selected 9 circRNAs which were not only correlated with cancer developments but also associated with adipocyte metabolism(ciRS-7, circBANP, circ-0104689, circ-0000190, circ-0001895, circ-0004277, circ-001569, circ-0001649, circ-100,876) to detect their expressions and stabilities in breast cancer cells by using RT-qPCR and electrophoresis. Only circ-0000190 was detected to be stable. The circRNA hsa_circ_0000190, also known as circCNIH4, has been proven to serve as a potential non-invasive diagnostic biomarker for gastric cancer [24]. The functional experiments then confirmed correlation between miR-135b and circCNIH4. These findings suggested that miR-135b-circCNIH4 might be important molecular targets for novel therapies of obesity-related cancers .

Therefore, we hypothesized that adipocytes might accelerate the progression of breast cancer by modulating FOXO1/miR-135b/circCNIH4 axis through EMT pathway.

## Materials and methods

### Patients and tissues

The experiment was in accordance with the ethical guidelines of the Helsinki Declaration and was approved by the Human Ethics Committee of The First Affiliated Hospital with Nanjing Medical University(acceptance no.: 2019-SRFA-048 and 2021-SR-131). All patients have got written informed consents.

We retrospectively screened 383 patients with breast diseases diagnosed in the first affiliated hospital with Nanjing Medical University from January 2020 to December 2020. After comprehensive skimming, 75 patients were expelled for high pressure, diabetes, hyperlipidemia, endocrine diseases, breast surgery history, total hysterectomy history. Then patient’s height, weight, TC, TG, HDL-C, LDL-C, clinical pathology information were collected (Supplementary Table 1 (Cohort 1)).

Then we collected 20 breast cancer patients diagnosed from January to September 2022 and retrieved their paired breast cancer tissues and normal breast tissues, instantly stored in liquid nitrogen (Supplementary Table 4 (Cohort 2)).

### Cell culture

The MCF-7(RRID: CVCL_0031), ZR-75-1(RRID: CVCL_0588), MDA-MB-231(RRID: CVCL_0062), Hs578T(RRID: CVCL_0332) and SK-BR-3(SkBr3)(RRID: CVCL_0033) human breast cancer cell lines and the Hpa-V(RRID: CVCL_UR30) adipose cell line were purchased from the Cell Bank of the Chinese Academy of Sciences (Shanghai, China). The SUM1315MO2(SUM1315)(RRID: CVCL_5589) breast cancer cell lines were kindly provided by Professor Stephen Ethier (University of Michigan, AnnArbor, MI, USA). All cell lines were cultured in DMEM(MDA-MB-231, Hs578T, SUM1315, SkBr3 and Hpa-V) or RPMI 1640(MCF-7, ZR-75) medium (Kaiji, Nanjing, China) supplemented with 10% fetal bovine serum (Gibco), 100 µg/ml streptomycin and 100 U/ml penicillin in a humidified incubator at 37 ℃ in an atmosphere of 5% CO_2_.

To perform cell co-culture experiments, we used a 6-well transwell chamber with pore size of 0.4 mm(Corning). Then 1mL serum-free DMEM medium including approximately 4 × 10^4^ Hpa-V cells were seeded in the upper chamber, while 5 × 10^5^ MDA-MB-231 or SkBr3 cells with 10% FBS DMEM medium were added into the lower chamber. The co-cultured group were named MDA-MB-231/Hpa-V or SkBr3/Hpa-V. After 48 h, MDA-MB-231/Hpa-V, SkBr3/Hpa-V on the lower chamber and the control group cells were gathered to achieve the following studies.

### Sequencing of mRNA expression profiles

Total RNA was extracted from MDA-MB-231/Hpa-V and MDA-MB-231 cells by using a miRNeasy Mini Kit (Qiagen, Hilden, Germany) following the manufacturer’s instructions. The RNA concentrations and purities were measured at 260/280 nm by using a NanoDrop 2000 spectrophotometer (Thermo Electron Corporation, USA) and by Bioanalyzer 4200 (Agilent, santa Clara, CA, USA). The NGS libraries were prepared using HISAT2 for Illumina^®^ (Vazyme, Nanjing, China) and obtained most of the transcripts in this state. The sequencing results were published by previous study [25] and shared to sequence read archive(SRA)(The BioProject accession: PRJNA668527).

### Differential expressions of mRNAs

The mRNAs with a P value < 0.05 and |log2 (fold change)|>3 were considered differentially expressed by analysis of DEseq2. The mRNAs with no expression in neither samples were eliminated.

### Gene ontology and KEGG pathway analysis

The selected mRNAs were further analyzed with Gene Ontology (GO) enrichment analysis and the Kyoto Encyclopedia of Genes and Genomes (KEGG) pathway through Metascape database (https://metascape.org/gp) to investigate their functions. P value < 0.05 was considered statistically significant.

### Total RNA extraction and quantitative real-time polymerase chain reaction (RT-qPCR)

RNA from cells and tissues were subsequently isolated using the RNA simple total RNA Kit (TIANGEN, Beijing, China) in line with the manufacturer’s protocols and RNA was quantified by Nanodrop 2000 spectrophotometer (Thermo Scientific, USA). Then, 500 ng of cellular RNA were reverse transcribed to cDNA by using the PrimeScript™ RT master mix (TaKaRa, Dalian, China) on an Eppendorf AG (Hamburg, Germany) with 37 ℃ 15 min following by 85 ℃ 5s. Specific divergent primers of circRNA and specific primers of *FOXO1* were used to quantify the amount of circCNIH4 and *FOXO1* mRNA, internal β-actin as the internal control. Stem-loop method was used to quantify the amount of miRNA and small nuclear U6B (RNU6B) RNA was used as an internal standard. The amplification of cDNA was done on a StepOnePlus™ Real-Time PCR System (Roche, Australia) by using SYBR (Beyotime, Shanghai, China) with 40 cycle reactions. All reactions were run in triplicates.

### Fluorescence in situ hybridization (FISH) assay

We ordered sets of fluorescent FISH probe mix and internal control probes from commercial sources (RIBOBIO, Guangzhou, China). The pooled FISH probes were resuspended to a final concentration of 20 µM in RNase-free storage buffer and were stored away from light at −20 ℃. The FISH assay was performed according to the manufacturer’s protocol. Finally, cells photographed by applying a microscope (Carl ZEISS, USA).

### Overexpression plasmid construction

The plasmid overexpressing circCNIH4 was constructed using circBasicTM (HANBIO, Shanghai, China) according to the manufacturer’s instructions.

### Transfection experiment

MiR-135b mimics/inhibitors, negative control of mimics/inhibitors(mimics/inhibitors-NC), plasmic-FOXO1(pFOXO1), plasmic-circCNIH4(OE-circCNIH4), negative control of plasmic(OE-NC) were synthesized (RIBOBIO, Guangzhou, China). 2 × 10^5^ cells were planted in each of the 6-well plate followed by cell density reaching 50% fusion after 24 h. Then cells were transfected by the mixture of mimics/mimics-NC/inhibitors/inhibitors-NC/OE-circCNIH4/OE-NC/pFOXO1(10nM) and 100 μL Opti (Gibco) with 7.5 μL lipoRNAiMAX (Invitrogen) and 100uL Opti composition. After transfection, the cells were incubated with 1.8 mL appropriate medium for 24–48 h before applied to other experiments.

### Luciferase reporter assay

PmirGLO Dual luciferase vectors (Promega, Madison, WI, USA) were used for construction of dual luciferase reporter plasmids for luciferase reporter assay. Sequences of circCNIH4 and its mutated type were separately cloned into the vectors, called PmirGLO-circCNIH4-W and PmirGLO-circCNIH4-M. MCF-7 and MDA-MB-231 cells were transfected with 8 µg PmirGLO-circCNIH4-W or PmirGLO-circCNIH4-M, along with 1.6 µM miR-135b mimics. After 24 h, luciferase activity was evaluated using the dual-luciferase reporter kit (Promega, Madison, WI, USA). The relative firefly luciferase activity was normalized to Renilla luciferase activity.

###  Wound healing

First, co-cultured and transfected SkBr3 or MDA-MB-231 cells and their parent cells in 6-well plates were cultured to 90–95% confluency. Then a linear wound was scraped by a sterile 200 µl Pipette head across the confluent cell layer. Cells were washed thrice by PBS to remove floated cells and debris. Finally, the width of wounds was photographed by camera (Canon, Japan) instantly, 24 h and 48 h later.

###  Migration assays

About 5 × 10^4^ cells were seeded in the upper chamber of transwell assay inserts (pore size of 8 mm; Corning) with 200 µL serum-free DMEM medium, and 600 µL DMEM medium containing 20% FBS was added into the lower chamber by a 24-well transwell chamber. Then cells on the filter surface were fixed with methanol before stained with crystal violet. Photographs were taken with a microscope(Carl ZEISS, USA) after 24 h and 48 h. The cell numbers were calculated in three random fields for each chamber.

### Invasion assays

Before seeding cells in upper transwell chamber, 100 µL mixture (1:8) of Matrigel (Coring, Coring, NY, USA) and serum-free DMEM medium(Kaiji, Nanjing, China) were coated on the upper transwell chamber. After 4 h for Matrigel concretion, the followed steps were in accordance with migration assays.

### Western blot

Total proteins were collected from cells 48 h after transfection following harvested using a RIPA lysis buffer(Biouniquer Technology, Nanjing, China) based on the manufacturer’s instruction. Then the purity and quality of proteins were measured with BCA protein assay kit(Beyotime, China). Equal amounts of proteins were separated by electrophoresis using 8% sodium dodecyl sulfate (SDS) polyacrylamide gels before transferring to polyvinylidene difluoride membranes (Sigma, Germany). The membranes were blocked in 5% skim milk for 2 h and then probed with primary antibodies against FOXO1 (1:1000; Cell Signaling Technology(CST), China) E-cadherin(1:1000; CST, China), vimentin(1:1000; CST, China), N-cadherin(1:1000; CST, China) or GAPDH (1:10000; Proteintech, America) at 4 °C overnight, followed by the secondary antibody (1:1000; Beyotime, China) for 1 h. Finally, after washing, enhanced chemiluminescence (ECL) plus kit (Millipore, America) was applied for visualization and GAPDH was utilized as an internal control.

### Immunohistochemistry

The Immunohistochemistry(IHC) staining of paraffin-embedded breast tissues was accomplished in accordance with protocols. The primary anti-FOXO1 antibody(1:50; Cell Signaling Technology, China) was used.

Also, FOXO1 was confirmed with use of breast cancer tissue microarrays by Outdo Biotech(Shanghai, China) containing 85 paired breast cancer and peritumoral tissue samples (Supplementary Table 5 (Cohort 3).

The IHC IRS scores were performed by two individual pathologists blinded to the clinical data.

### Animal experiments

The animal studies were approved by the Animal Care and Use Committee of Nanjing Medical University (acceptance no.: IACUC-1,906,022 and IACUC-2,104,027) and complied with the guidelines of the National Institutes of Health.

Female BALB/c nude mice(8–10 weeks old) were randomly divided into two groups. We gently injected 5 × 10^5^ MDA-MB-231 cells stably overexpressing circCNIH4 (circCNIH4) and their control vectors (circ-NC) (constructed by Genechem, Shanghai)into the tail vein by using an insulin syringe. Then mice were fostered in an air-filtered and pathogen-free condition. After 12 weeks, all mice were intra-peritonelly injected 200 μL (20 mg/mL) D-Luciferin Firefly potassium salt (Sciencelight, China) and imaging by IVIS Spectrum (PerkinElmer, America).

###  Bioinformatic analysis

The miRNAs targeted gene(*FOXO1*) was predicted using the TargetScan, PicTar and miRDB software. The circRNAs binding to miR135b was predicted by ENCORI (https://rnasysu.com/encori/index.php). The target of circCNIH4 was predicted by software circPrimer (http://www.bioinf.com.cn/) [26]. Further correlation between patients survival and miRNA/FOXO1 expression was determined through analysis of The Cancer Genome Atlas database (http://cancergenome.nih.gov/), UALCAN (http://ualcan.path.uab.edu/) and Kaplan-Meier Plotter (http://kmplot.com/).

### Statistical analysis

Statistical analysis was performed using SPSS (23.0 vision) and GraphPad 8.0, one-way ANOVA and Student’s t-test were used. P value < 0.05 was considered as significant difference. All experiments were carried out in triplicates.

### Data and software availability

https://www.ncbi.nlm.nih.gov/sra/(PRJNA668527).

https://data.mendeley.com/datasets/f59h4c6fj7/draft?a=67e5af78-f1c4-4979-9a8d-48126fc2ea0a.

## Results

### Obesity related to breast cancer incurrence and development

**Fig. 1:**
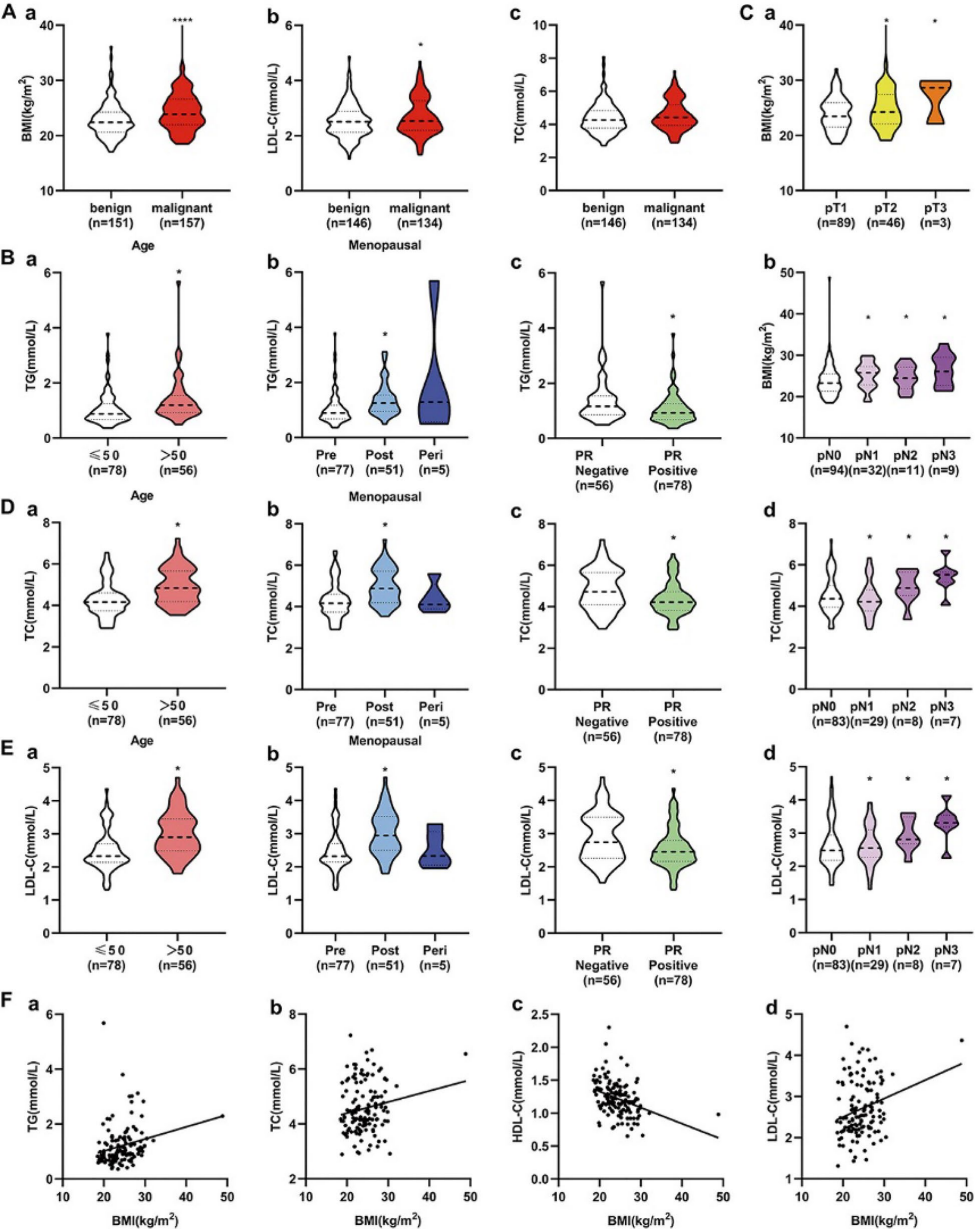
Clinical information of BMI, TC, TG, LDL-C, HDL-C in breast disease patients(Cohort 1). **A** The levels of BMI, LDL-C, TC in breast malignant and benign patients; **B–E** The level of TG, BMI, TC, LDL-C in subgroups of breast cancer patients; **F** Correlation curves between BMI and TG (R^2^=0.05241, P=0.0078), TC (R^2^=0.02744, P=0.0558), HDL-C (R^2^=0.1249, P＜0.0001), LDL-C (R^2^=0.06178, P=0.0038). *P＜0.05.

BMI and LDL-C concentration were significantly higher in malignant than in benign breast patients. TC was higher in malignant with *P* = 0.057 (Fig. 1A). In subgroup analysis, TG was related to age, menopause and PR receptor (Fig. 1B). BMI was associated with T and N status (Fig. 1C). Moreover, TC (Fig. 1D) and LDL-C (Fig. 1E) concentration were related to age, menopause, PR receptor and N status. Simultaneously, BMI was positively correlated with TC, TG, and LDL-C concentration with significant differences, while obviously negatively correlated with HDL-C concentration (Fig. 1F).

### Adipose cells promote invasion and migration of breast cancer cells by EMT pathway

**Fig. 2:**
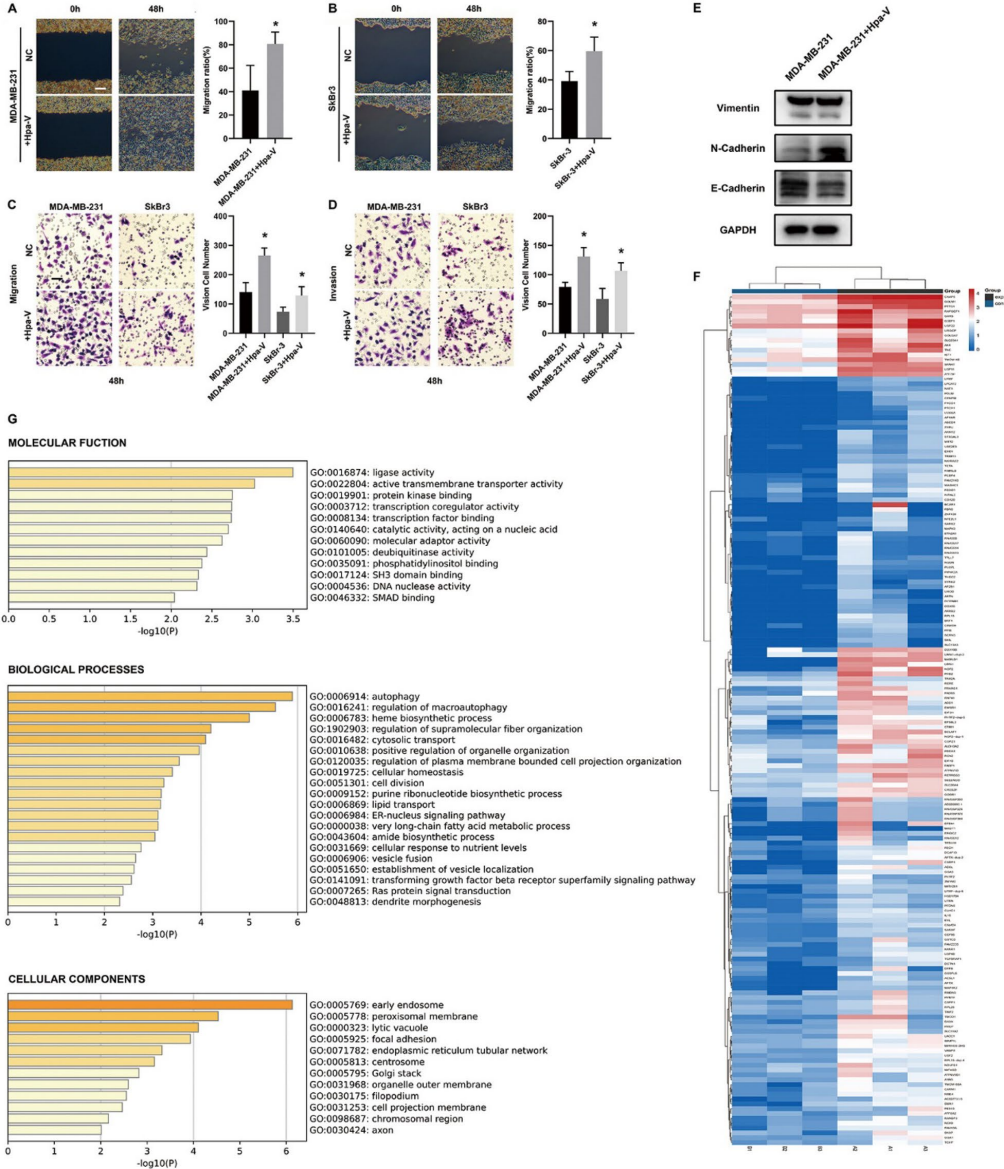
Adipocytes promote breast cancer cell invasion and migration through EMT pathway and the transcripts sequencing results.** A**, **B** Would healing test; Bar: 250um; **C–D** Transwell migration and Matrigel invasion tests; Bar: 50um; **E** Western Blot of EMT pathway in breast cancer cells; **F** Heatmap of differential expressed transcripts in MDA-MB-231/Hpa-V and MDA-MB-231 cells; **G** GO pathway analysis. *P＜0.05

A fixed 4 × 10^4^/5 × 10^5^ cell proportion(Hpa-V/breast cancer cells) co-cultured for 48 h was applied in the following experiments. Wound healing (Fig. 2A–B) and transwell migration assays (Fig. 2C–D) showed that the migration ability of MDA-MB-231/Hpa-V and SkBr3/Hpa-V cells were increased compared with the control cells. Matrigel invasion experiments concluded that MDA-MB-231/Hpa-V and SkBr3/Hpa-V cells obtained higher invasiveness compared with the control group(Fig. 2C–D). From western blots, we learned that N-cadherin was upregulated in MDA-MB-231/Hpa-V compared with MDA-MB-231 cells, while E-cadherin was downregulated, although vimentin showed no obvious difference(Fig. 2E). The results were in accordance with the confirmation by Hua et al [13].

### Adipose decreased FOXO1 in breast cancer cells

By further analyzing the mRNA sequencing results in MDA-MB-231/Hpa-V and MDA-MB-231 cell, the heatmap showed that 176 transcripts were upregulated in MDA-MB-231/Hpa-V than MDA-MB-231 cells (Fig. 2F, P<0.05). We abandoned the transcripts without detection of transcripts expressions in neither of the samples in both groups. Then we selected log2counts larger than 3. GO pathway analysis presented that the genes were mainly enriched in ligase activity, active transmembrane transporter activity, protein kinase binding, transcription coregulator activity and transcription factor binding by molecular function, autophagy, regulation of macroautophagy, heme biosynthetic process by biological processes, early endosome, peroxisomal membrane, lytic vacuole, focal adhesion by cellular components (Fig. 2G, P<0.01). Then we found the expression of FOXO1 was stable.

**Fig. 3:**
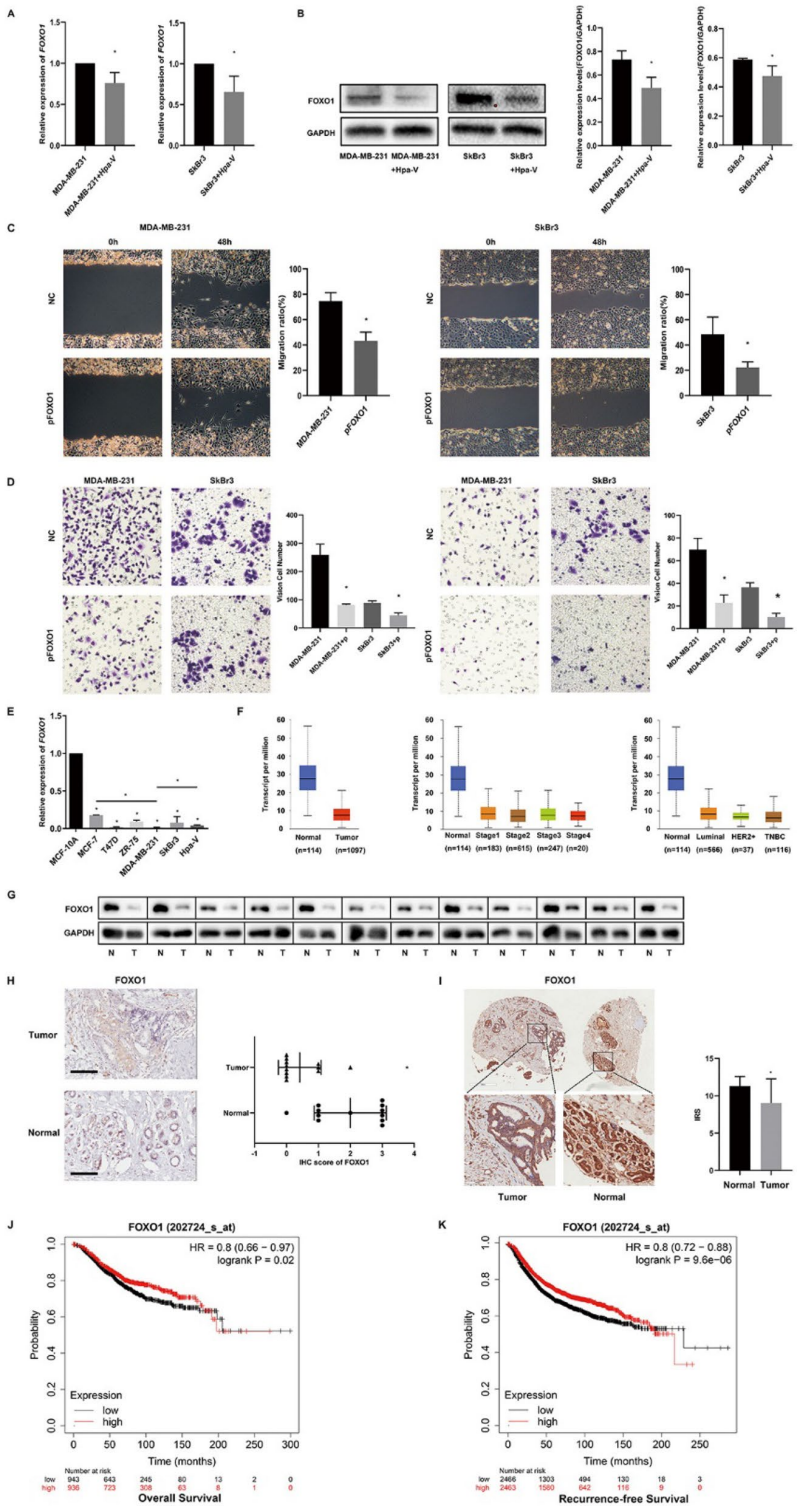
Adipocytes promote breast cancer cell invasion and migration through FOXO1 and the characteristics of FOXO1. **A** Relative expression of *FOXO1* mRNA levels by RT-qPCR; **B** Relative expression of FOXO1 protein levels by Western Blot;** C** Would healing test; Bar: 250 μm; **D** Transwell migration and Matrigel invasion tests; Bar: 50 μm; **E** Relative expression of *FOXO1* mRNA level in subtypes of breast cancer cells; **F** The expression of FOXO1 in normal breast tissues and breast cancer tissues and subgroups from TCGA dataset; **G** The protein levels of FOXO1 in 12 out of 20 paired tumor and normal breast tissues(Cohort 2); **H** The immunohistochemistry of FOXO1 in tumor and normal breast tissues (Cohort 2); Bar: 100 μm; **I** FOXO1 expression and IRS scores in tissue microarrays of 85 paired breast cancer and peritumor tissues (Cohort 3);** J** Kaplan-Meier survival curves of FOXO1 expression and Overall survival in breast cancer patients; **K** Kaplan-Meier survival curves of FOXO1 expression and Recurrence-free survival in breast cancer patients. * P＜0.05

After coculturing with Hpa-V cells, the mRNA and protein levels of FOXO1 in breast cancer cell lines were suppressed (Fig. 3A, B). To identify the effect of FOXO1 on breast cancer cells, we constructed specific plasmid overexpressing FOXO1, then separately transfected the p-FOXO1 into the MDA-MB-231 and SkBr3 cells. After 48 h, the overexpression of FOXO1 was confirmed to significantly damage the migration of MDA-MB-231 and SkBr3 cells by the wound healing assays, comparing to NC group (Fig. 3C). Transwell and matrigel assays showed that the migration and invasion of MDA-MB-231 and SkBr3 cells were weakened by the overexpression of FOXO1 (Fig. 3D).

### Relative expression of FOXO1 in breast cancer

In diverse cell lines, the basic levels of *FOXO1* were lower in breast cancer cell lines and Hpa-V cells than normal breast cells (Fig. 3E). And its expression in more aggressive type breast cancer cells were lower than less aggressive cells.

We analyzed TCGA dataset of 1211 breast cancer patients. The results conveyed that the expression of FOXO1 was obviously lower in breast cancer tissues than normal breast tissues. In subgroup analysis, the expression level of FOXO1 was lower in stage2 than stage1, lower in 61–80 year old than 41–60 year old, lower in TNBC than Luminal, lower in post-menopausal (Fig. 3F). Meanwhile, we examined 20 paired tumor and normal breast tissues, but only 12 pairs were accessible and found that the protein levels of FOXO1 were lower in tumor than normal tissues by western blot and IHC (Fig. 3G–H, Supplementary Table 4 (Cohort 2)). This result was validated by tissue microarrays (Fig. 3I, Supplementary Table 5 (Cohort 3)).

To further evaluate the potential relationship of FOXO1 expression with patient survival, we generated Kaplan-Meier survival curves and found that lower FOXO1 expression informed poorer OS (Fig. 3J; HR = 0.8, 95% CI 0.66–0.97, *P* = 0.02) and RFS (Fig. 3K; HR = 0.8, 95% CI 0.72–0.88, *P*<0.01).

### FOXO1 is one of target genes of miR-135b

**Fig. 4:**
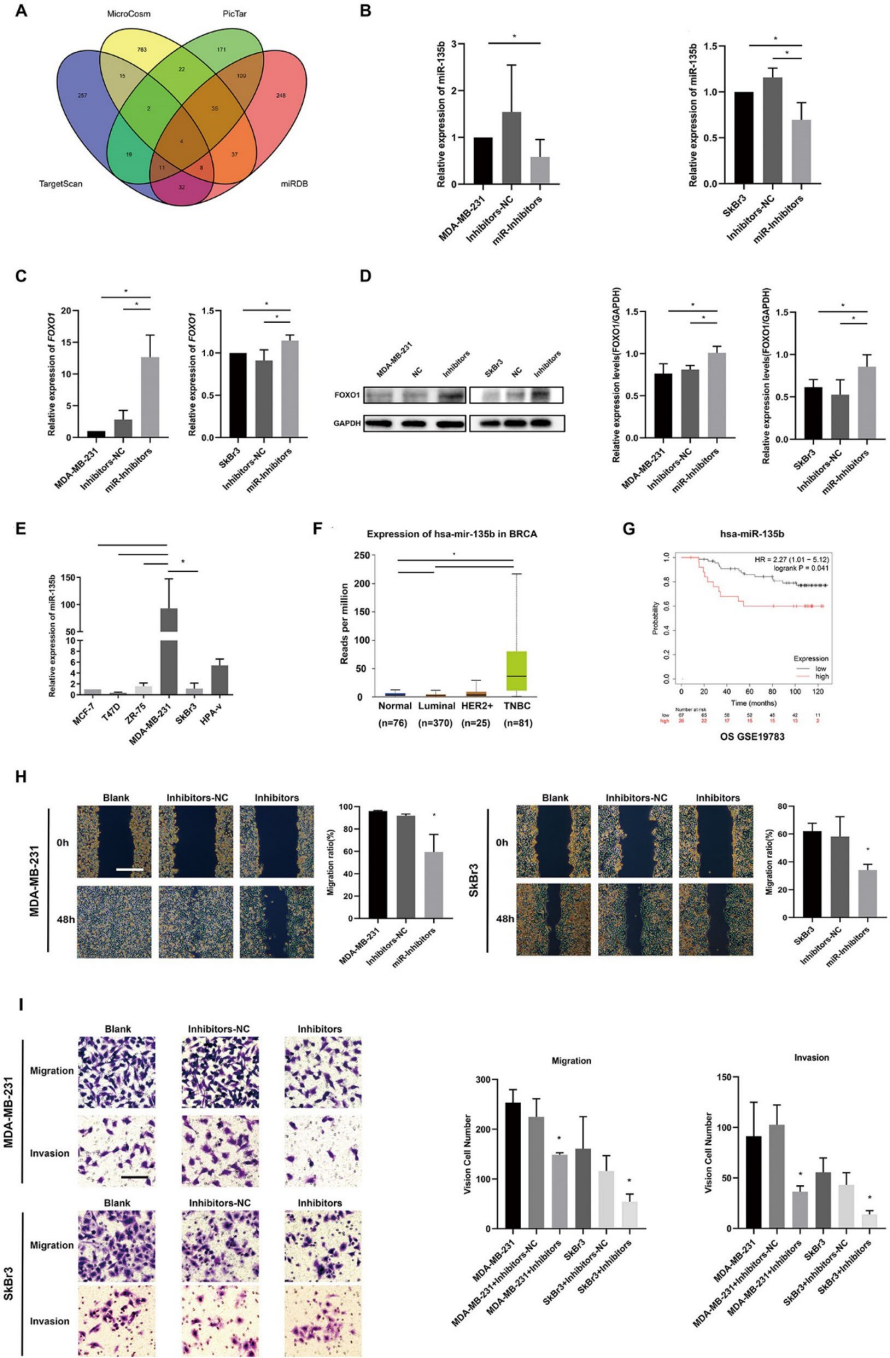
FOXO1 is a downstream target of miR-135b and miR-135b promotes metastasis of breast cancer cells. **A** Predicted target genes of miR-135b; **B** Effects of miR-135b inhibitors; **C** Relative expression of *FOXO1* mRNA level after inhibiting miR-135bs; **D** Western Blot of FOXO1 protein levels in breast cancer cells after inhibiting miR-135b;** E** Relative expression of mir-135b in diverse subtypes of breast cancer; **F** The expression of miR-135b in subtypes of breast cancer from TCGA database;** G** Overall survival curves of breast cancer in GSE19783;** H** Wound healing tests; Bar: 250 μm; **I** Transwell and Matrigel tests; Bar: 50um. *P＜0.05

The *FOXO1* was predicted as one targeted gene of miR-135b by searching the TargetScan, PicTar, miRDB and MicroCosm software (Fig. 4A). Then we transferred inhibitors of miR-135b in two different types of breast cancer cell lines(MDA-MB-231 and SkBr3). After transfection, the expression of miR-135b was apparently depressed than their parental cells (Fig. 4B). After inhibiting the levels of miR-135b, the mRNA and protein levels of FOXO1 were significantly downregulated through RT-qPCR (Fig. 4C) and western blot (Fig. 4D) in MDA-MB-231 and SkBr3 cells.

Then we detected the expression of miR-135b in diverse subtype breast cancer cells, concluding that miR-135b was stably and obviously higher expressed in MDA-MB-231 than other cell lines (Fig. 4E). Simultaneously, we detected that the expression of miR-135b was higher in TNBC than Luminal subtype breast cancer from TCGA database (Fig. 4F). From KMPlotter survival curves, miR-135b was a negative biomarker for overall survival of breast cancer in GSE19783 (Fig. 4G).

The wound healing, transwell and matrigel assays were adopted to unearth the migrative and metastatic ability of breast cancer cells after the inhibition of miR-135b. The results displayed that when we inhibited the expression of miR-135b in MDA-MB-231 and SkBr3, the migration and metastasis of cells were reduced (Fig. 4H–I).

### CircCNIH4 acts as a miRNA sponge for miR-135b

**Fig. 5:**
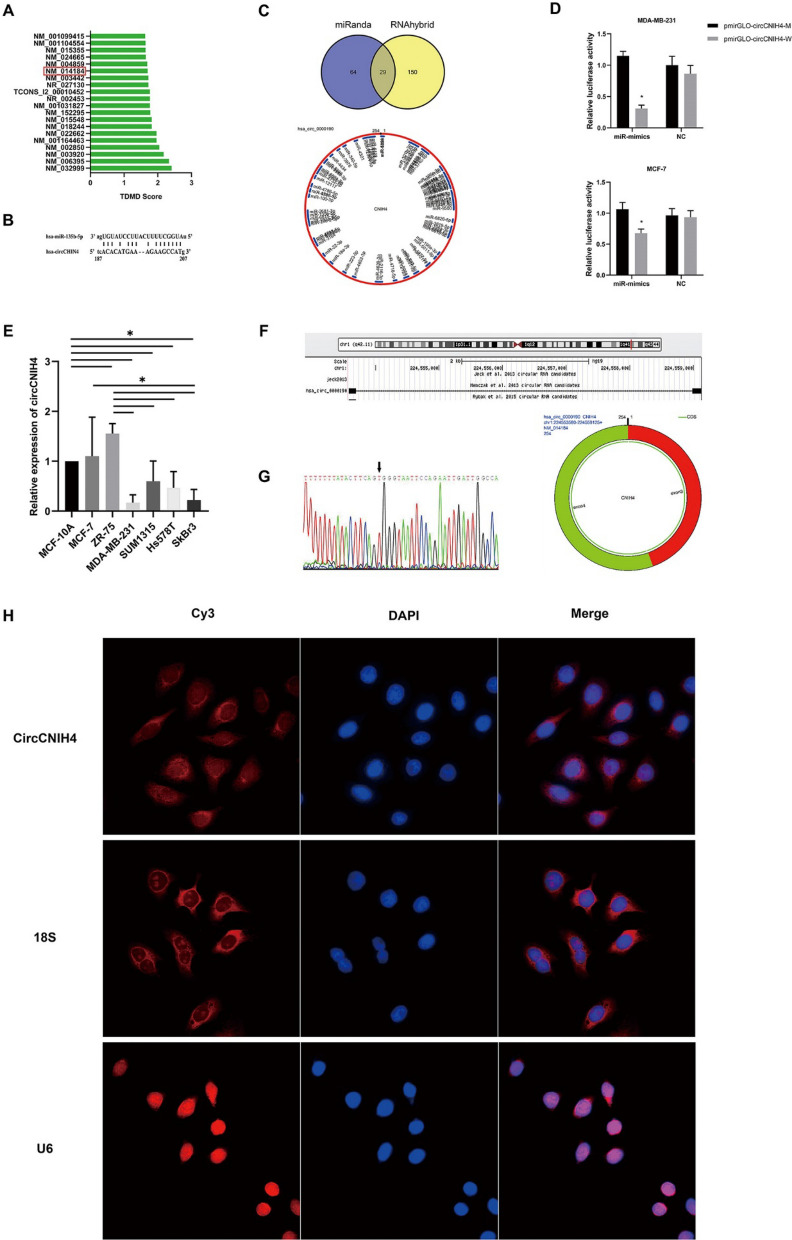
CircCNIH4 binds to miR-135b and the characteristics of circCNIH4. **A** Predicted binding circRNAs to miR-135b; **B** Binding sites of circCNIH4 and miR-135b; **C** Predicted downstream target miRNAs of circCNIH4; **D** Dual-luciferase reporter assay; **E** Relative expression of circCNIH4 in different subtypes of breast cancer cells and breast epithelial cells; **F** Chromosomal region of circCNIH4 from circBase database; **G** CircCNIH4 locates in exon3 and exon4 of cornichon family AMPA receptor auxiliary protein 4, and the Sanger sequence confirms the junction of cricCNIH4; **H** Location of circCNIH4 in breast cancer cells by FISH tests. *P＜0.05

The ENCORI website predicted circRNAs binding to miR135b (Fig. 5A). We picked up 9 known circRNAs to detect their expression levels in breast cancer cells using RT-qPCR. Only circ-0000190(circCNIH4) was detected to be stable in the breast cancer cells by electrophoresis (Fig. S1A, B, Supplementary Table 2). CircCNIH4 was a superior choice for its higher and stable level and contained complementary binding sites with miR-135b (Fig. 5B). MiRanda (August 2010 Release) and RNAhybrid were used to predict downstream target miRNAs of circCNIH4 (Fig. 5C). MiR-135b was a superior choice, which in turn confirming the relation between circCNIH4 and miR-135b. Also, Dual-luciferase reporter assay showed that the co-transfection of PmirGLO-circCNIH4-W and miR-135b mimics remarkably decreased the luciferase activity compared to PmirGLO-circCNIH4-M in two subtypes of breast cancer cells. Therefore, these results implied that circCNIH4 could harbor miR-135b (Fig. 5D).

To confirm the fundamental expression of circCNIH4 in different breast cancer cells and breast epithelial cells, RT-qPCR was performed. Apparently, circCNIH4 was decayed in aggressive cancer cells than normal cells. Also, the results appeared that the expression of circCNIH4 in estrogen receptor positive breast cancer cell lines(MCF-7, ZR-75) were significantly upregulated comparing to aggressive triple negative breast cancer cells(MDA-MB-231, SUM1315, Hs578T) and her-2 positive breast cancer cell line (SkBr-3) (Fig. 5E).

According to the circBase database, circCNIH4 is generated from chromosomal region 1q42.11 (chr1:224553581–224,559,125) (Fig. 5F), whose relative gene is located in exon3 and exon4 with the gene name of cornichon family AMPA receptor auxiliary protein 4 (CNIH4) .Sanger sequence has confirmed the sequence of the junction of circCNIH4 (Fig. 5G) [27]. Fluorescence in situ hybridization (FISH) assay was carried out to investigate the localization of circCNIH4 in the MDA-MB-231. The result showed that the majority of circCNIH4 was located in the cell cytoplasm (Fig. 5H).

### CircCNIH4 suppresses invasion and migration of breast cancer cells

**Fig. 6:**
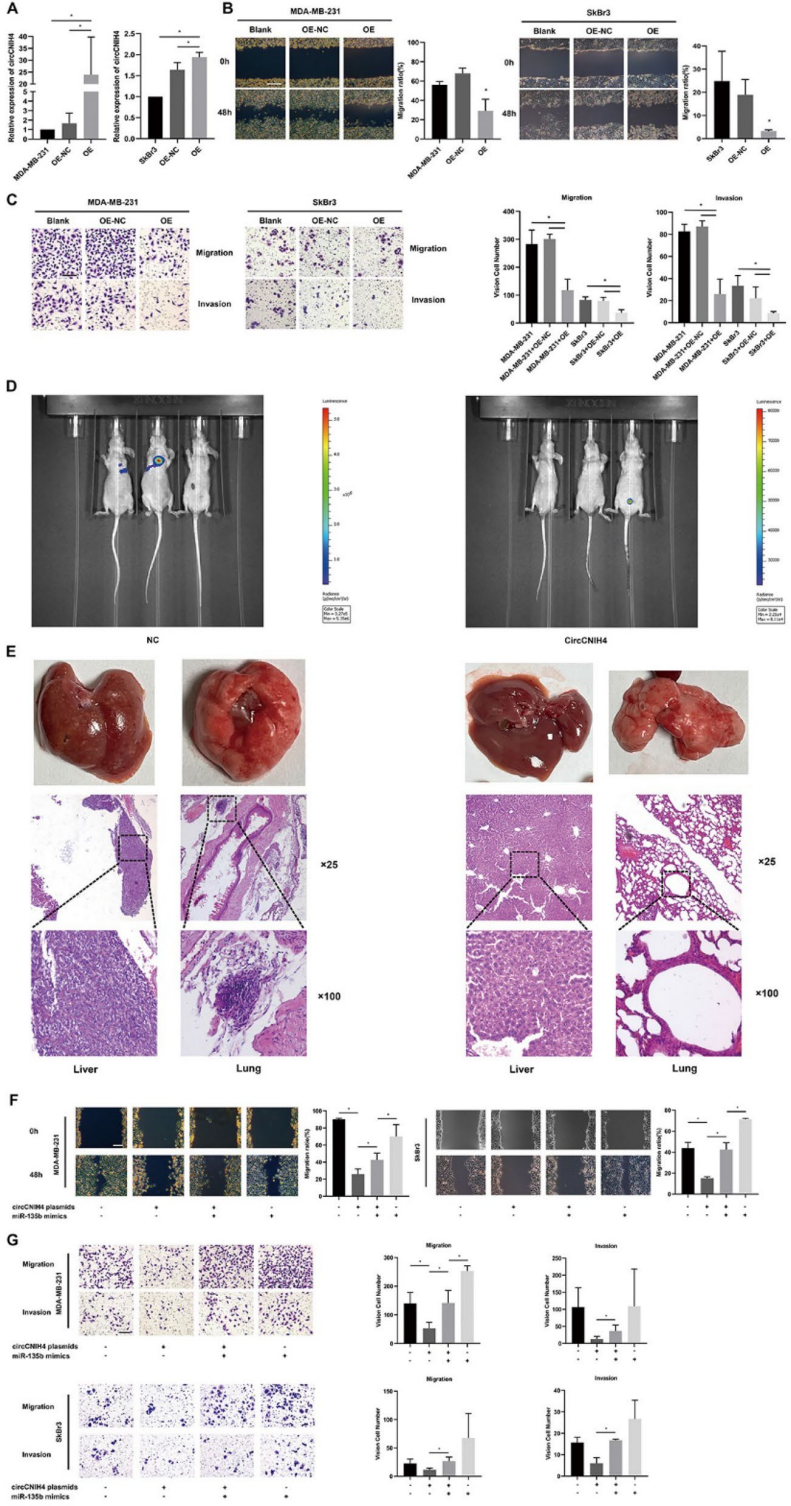
CircCNIH4 suppresses invasion and migration of breast cancer cells. **A** Relative expression of circCNIH4 after transfected overexpressing plasmids; **B** Wound healing tests and migration ratio; Bar: 250 μm; **C** Transwell and Matrigel assays; Bar: 50 μm; **D** Imaging of metastatic areas *in vivo* after injected MDA-MB-231 cells with virus constantly overexpressing circCNIH4 or negative control; **E** Images and IHC of liver, lung metastatic sites; **F** Rescue Wound healing assays; Bar: 250 μm; **G** Rescue Transwell and Matrigel tests; Bar: 50 μm. *P＜0.05

To identify the effect of circCNIH4 on breast cancer cells, we constructed specific plasmid overexpressing circCNIH4, then separately transfected the p-circCNIH4(OE) or blank plasmid (OE-NC) into the MDA-MB-231 and SkBr3 cells. After 48 h, the expression levels of circCNIH4 in cells transfected with OE-circCNIH4 were obviously upregulated in contrast with OE-NC (Fig. 6A). Also, the overexpression of circCNIH4 was confirmed to significantly damage the migration of MDA-MB-231 and SkBr3 cells by the wound healing assays, comparing to NC group (Fig. 6B). Transwell and matrigel assays showed that the migration and invasion of MDA-MB-231 and SkBr3 cells were weakened by the overexpression of circCNIH4 (Fig. 6C). In vivo, stable overexpression of circCNIH4 lead to impaired metastatic ability (Fig. 6D, E).

In rescue experiments, the metastasis and invasion of cells transfected with circCNIH4 plasmids and miR-135b mimics were reversed in comparison with cells transfected with single circCNIH4 plasmids or miR-135b mimics through wound healing assays (Fig. 6F), transwell assays and matrigel assays (Fig. 6G) obviously.

### Adipocytes affected FOXO1/miR-135b/circCNIH4 through EMT pathway and copper homeostasis

**Fig. 7:**
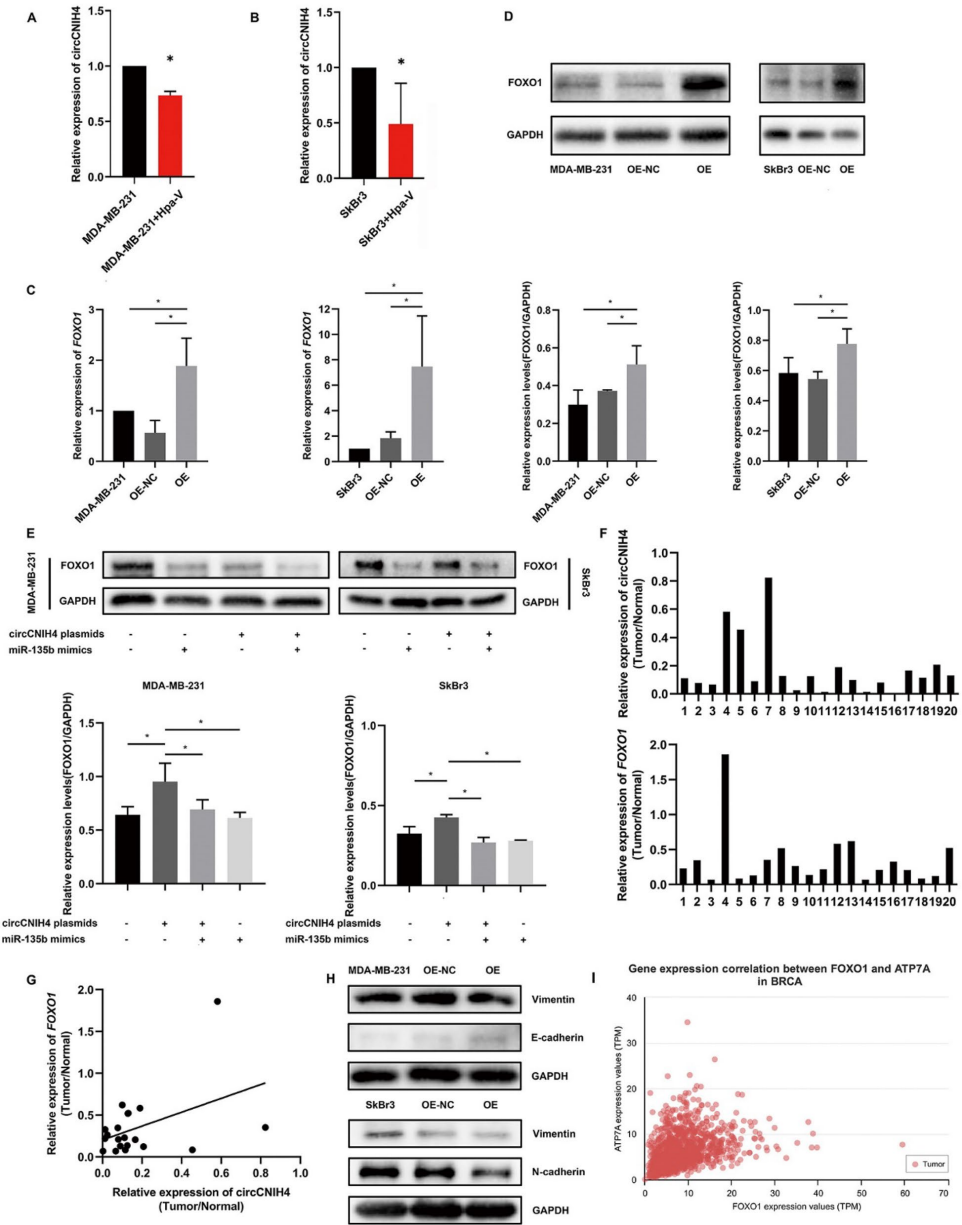
Adipocytes promote metastasis of breast cancer by FOXO1, EMT pathway and regulating copper homeostasis. **A, B** Relative expression of circCNIH4 in MDA-MB-231 and SkBr-3 after co-cultured with Hpa-V cells and their parental cells; **C** Relative expression of *FOXO1* mRNA levels by RT-qPCR; **D** Relative expression of FOXO1 protein levels by Western Blot; **E** Western Blot in rescue experiments; **F** Relative expression of circCNIH4 and *FOXO1* mRNA levels by RT-qPCR in breast cancer tissues versus normal tissues(Cohort 2); **G** Correlation curves between expression of circCNIH4 and *FOXO1* mRNA levels (R^2^=0.1896, P=0.0275); **H** Western Blot of EMT pathway in breast cancer cells; **I** Pearson correlation between FOXO1 and ATP7A. *P＜0.05.

After cocultured with Hpa-V cells, the expression of circCNIH4 in MDA-MB-231/Hpa-V and SkBr3/Hpa-V was down-regulated to 0.73 and 0.49 compared to MDA-MB-231 and SkBr3 breast cancer cell line respectively (Fig. 7A, B).

Then via RT-qPCR and western blot, we unearthed that the mRNA and protein level of FOXO1 was in consistence with overexpression of circCNIH4 in MDA-MB-231 and SkBr3 (Fig. 7C, D). Also, the protein expression of FOXO1 in cells transfected with circCNIH4 plasmids and miR-135b mimics was lower than cells with only circCNIH4 overexpression (Fig. 7E). In 20 paired tumor and normal breast tissues, the levels of circCNIH4 and FOXO1 were lower in tumor than normal tissues (Fig. 7F). In breast cancer tissues, the expression of circCNIH4 was positively related to FOXO1 expression by correlation curve (Fig. 7G). Simultaneously, in MDA-MB-231 cells, when overexpressing circCNIH4, vimentin was downregulated, while E-cadherin was upregulated. In SkBr3 cells, when overexpressing circCNIH4, vimentin and N-cadherin were downregulated (Fig. 7H). Besides, FOXO1 was supposed to participate in the copper homeostasis by regulating ATP7A (Pearson curve 0.33, *P*<0.05, Fig. 7I).

**Fig. 8:**
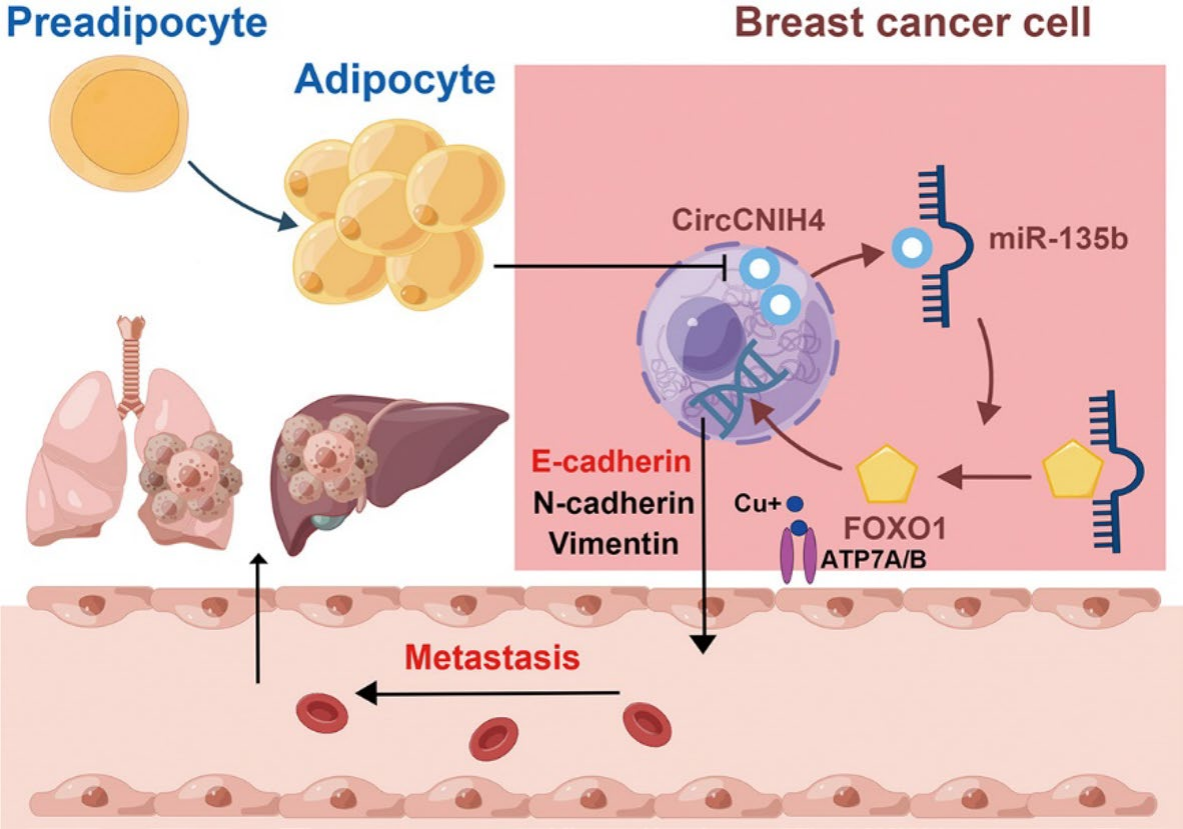
Mechanical diagram of adipocytes promote metastasis of breast cancer by attenuating the FOXO1 and circCNIH4 effects

The results appropriately supported our speculation that adipocytes stimulated breast cancer cell invasion and migration by FOXO1/miR-135b/circCNIH4 through EMT pathway and regulating copper homeostasis (Fig. 8).

## Discussion

Tumor development is associated with its surrounding microenvironment and obesity dramatically presents a hospitable environment to promoting tumors [4]. Also, obesity plays a critical part in breast cancer metastasis by generating an immune suppressive environment [28]. Detailed researches discovered that dyslipidemia interaction with USP9x and SMAD4 might govern TGF-β signaling during breast cancer metastasis [29]. In our clinical data, we summed up the fact that BMI as well as lipids concentration correlated the induction and development of breast cancer. Also, in vitro we detected that after co-cultured with adipocytes cells, breast cancer cells became more aggressive than their control cells, which was in consistent with the conclusion that adipocytes were able to improve the promotion of breast cancer.

We co-cultured adipocytes with breast cancer cells and then analyzed the differentially expressed mRNAs. GO pathway analysis presented that the genes were mainly enriched in ligase activity, active transmembrane transporter activity, protein kinase binding, transcription coregulator activity and transcription factor binding by molecular function. In general, FOXO1 regulated adipocyte differentiation through eliciting cell cycle arrest and upregulating the expression of p21 and p27 in a sigmoid activation pattern, which played an inhibitory role in the early period of adipocyte proliferation and the clonal expansion [30]. In breast cancer, decreased expression of FOXO1 has been linked to poor prognosis via interactions with the PI3K/Akt pathway, GATA3, and Annexin-1 expression [31]. Our study verified the depression of FOXO1 by adipocytes in vitro at the very first time. And we confirmed the tumor suppressive role of FOXO1 by analyzing its protein levels in paired breast cancer and normal tissues, and by examining the Kaplan-Meier survival curves of 1211 breast cancer patients from the TCGA dataset.

Through bioinformatic analysis and in vitro experiments, we identified FOXO1 as a downstream target of miR-135b and demonstrated that its expression was inhibited by adipocyte-induced miR-135b upregulation. Subsequently, we validated the oncogenic role of miR-135b in breast cancer through wound healing and transwell assays. Besides, miR-135b has been implicated in the lipolysis process of obesity [32]. In consequence, our results suggested that FOXO1 act as one target of miR-135b and inhibit the invasion and metastasis of breast cancer. At the same time, adipocytes might promote breast cancer development through the FOXO1/miR-135b axis.

Recent studies have highlighted the significance of circRNAs as competing endogenous RNAs (ceRNAs) in breast cancer. Studies have identified circEPSTI1 [33] and circIRAK3 [34] as independent prognostic markers for breast cancer. Numerous evidences had proved that circRNAs typically function as the miRNA sponges [35]. So with the help of ENCORI, MiRanda and RNAhybrid data, we found that miR-135b was the upmost related downstream target of circCNIH4, which was verified by dual-luciferase reporter assay. The latest literature has conveyed the potential role of circCNIH4 in cervical [36] and gastric cancer [37], and in adipose tissue metabolism [17, 38]. In this study, we focused on the novel role of circCNIH4 in adipocytes-related breast cancer.

To elucidate the underlying mechanism, we confirmed its expression in breast cancer cells treated with or without adipocytes by using RT-qPCR and electrophoresis. We further confirmed the characteristics of circCNIH4 in breast cancer cells through circBase database analysis, qRT-PCR, FISH test, wound healing and transwell assays. Our findings demonstrated that circCNIH4 functioned as a protective factor in breast cancer, mainly located in the cytoplasm. Moreover, the overexpression of circCNIH4 led to the attenuation of breast cancer cell invasion and metastasis both in vitro and in vivo.

Additionally, our analysis indicated a positive correlation between FOXO1 and circCNIH4 in breast cancer cells and tissues. We proposed that adipocytes promoted breast cancer invasion and migration through the FOXO1/miR-135b/ circCNIH4 axis.

EMT is a fundamental process that drives invasion and metastasis in various types of solid tumors, including breast cancer [39, 40]. In this study, we demonstrated for the first time that overexpression of FOXO1 could reverse the EMT process, leading to the suppression of metastasis in breast cancer. This finding provided strong evidence to support our hypothesis that FOXO1 had an anti-cancer role in breast cancer. Besides, FOXO1 was reported to be one of copper homeostasis related genes [41]. Therefore, our results suggested that adipocytes might modulate the FOXO1/miR-135b/ circCNIH4 axis, thus enhancing the malignancy of breast cancer by inducing EMT and regulating copper homeostasis in breast cancer cells.

Interestingly, Balaban and colleagues previously reported that adipocytes could transfer free fatty acids to breast cancer cells, promoting their proliferation and metastasis [42]. At the same time, adipocyte-derived exosomes have been suggested as a potential mediator of the link between obesity and cancer by delivering fatty acid oxidation [43]. However, further researches are required to elucidate the direct mechanisms by which adipocytes impact the characteristics of breast cancer cells.

## Conclusion

Therefore, we occasionally refreshed the association between obesity and breast cancer, highlighting the importance of managing body weight and maintaining a healthy lifestyle to reduce the risk of breast cancer. Besides, we also found that FOXO1 and circCNIH4 might have the potential as prognostic biomarkers for breast cancer patients due to the abilities to suppress the EMT pathway. This provides possibilities of novel therapies by promoting FOXO1 and circCNIH4 expressions to counteract the adverse effects of adipocytes on breast cancer.

### Supplementary Information

Additional file 1: Figure 1: Characterize of circCNIH4. (A): Relative expression of circRNAs; (B): Electrophoretic diagram. *P＜0.05; **P＜0.01. Table 1 Clinical information of breast patients (Cohort 1). Table 2 The list of primers of circRNAs for RT-qPCR. Table 3 The list of primers of miRNAs for RT-qPCR. Table 4 Clinicalpathology of 20 breast cancer patients (Cohort 2). Table 5 Clinicalpathology of breast cancer tissue microarrays (Cohort 3).

## Data Availability

Data is provided within the manuscript or supplementary information files.

## Declarations

### Animal studies

The animal studies were approved by the Animal Care and Use Committee of Nanjing Medical University (acceptance no.: IACUC-1906022 and IACUC-2104027) and complied with the guidelines of the National Institutes of Health.
