# Supplementary material for: Adipocytes promote metastasis of breast cancer by attenuating the FOXO1 effects and regulating copper homeostasis
Source: Cancer Cell Int. 2024 Aug 12;24:284. doi: 10.1186/s12935-024-03433-y (PMC11320833; doi:10.1186/s12935-024-03433-y)
Supplement: Supplementary file 1 — Additional file 1: Figure 1: Characterize of circCNIH4. (A): Relative expression of circRNAs; (B): Electrophoretic diagram. *P＜0.05; **P＜0.01. Table 1 Clinical information of breast patients (Cohort 1). Table 2 The list of primers of circRNAs for RT-qPCR. Table 3 The list of primers of miRNAs for RT-qPCR. Table 4 Clinicalpathology of 20 breast cancer patients (Cohort 2). Table 5 Clinicalpathology of breast cancer tissue microarrays (Cohort 3). [file 12935_2024_3433_MOESM1_ESM.docx]

**List of Supporting Information**

**Supplementary Figure 1**: Characterize of circCNIH4. **(A)**: Relative expression of circRNAs; **(B)**: Electrophoretic diagram. * P＜0.05; ** P＜0.01.


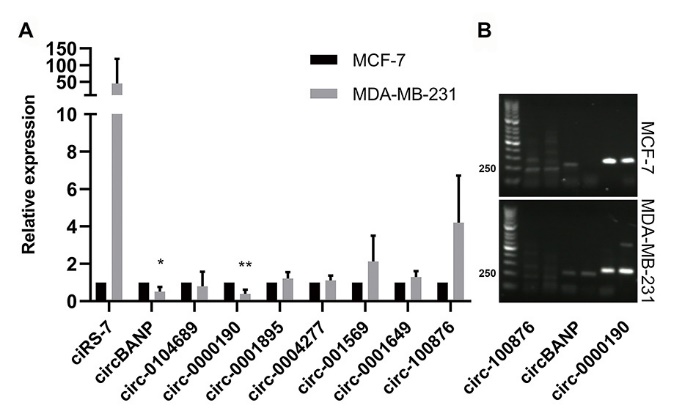


**Supplementary Table 1** Clinical information of breast patients(Cohort 1)

| Clinical information of breast patients | | |
| --- | --- | --- |
|  | benign | malignant |
| Number | 151 | 157 |
| Mean age(years) | 42 | 48 |
| Mean BMI(kg/m2) | 22.78 | 24.37 |
| Mean TC (mmol/L) | 4.36 | 4.56 |
| Mean TG (mmol/L) | 1.12 | 1.21 |
| Mean HDL-C (mmol/L) | 1.24 | 1.22 |
| Mean LDL-C (mmol/L) | 2.52 | 2.69 |
| Mean Lpa(mg/L) | 207.03 | 210.15 |
| pT |  | N(%) |
| 1(≤2cm) |  | 90(57.32) |
| 2(＞2cm,≤5cm) |  | 46(29.30) |
| 3(＞5cm) |  | 3(1.91) |
| pN |  |  |
| 0 |  | 94(59.87) |
| 1(≥1,≤3) |  | 32(20.38) |
| 2(≥4,≤9) |  | 11(7.00) |
| 3(≥10) |  | 9(5.73) |
| ER |  |  |
| negative |  | 48(30.57) |
| positive |  | 109(69.43) |
| PR |  |  |
| negative |  | 68(43.31) |
| positive |  | 89(56.69) |
| HER2 |  |  |
| negative |  | 111(70.70) |
| positive |  | 40(25.48) |
| Ki67 |  |  |
| ≤20% |  | 63(40.13) |
| ＞20% |  | 94(59.87) |
| M |  |  |
| negative |  | 153(97.45) |
| positive |  | 4(2.55) |

**Supplementary Table 2** The list of primers of circRNAs for RT-qPCR

| Name of circRNAs | Forward(5’-3’) | Reverse(5’-3’) |
| --- | --- | --- |
| hsa_circ_100876 | CTGGTGCAGTGGAAGCAGAG | CGACCCTCCATTGCTCTTCT |
| hsa_circ_BANP | CAGGACGGTCAGCGTCGT | GGCACAGCGTTGCTAATGAC |
| hsa_circ_0001895 | GCAAACAAGCAGGATCAGCAA | CCCATCCTTGCCCTTGGTAA |
| hsa_circ_0004277 | CACTTACAAGGCTTCCAC | CTTACTCAGCTCTGCTCC |
| hsa_circ_0000190 | ATACACAATCGAGGGCAGCT | CCAGTGCAATGACATGAGCA |
| has-ciRS-7 | TCAACTGGCTCAATATCCATGTC | ACCTTGACACAGGTGCCAT |
| hsa_circ_001569 | TCCCCTGAACATTCTCCCCAT | GAAAGCACTTGGTGAAGTCGG |
| hsa_circ_0001649 | AATGCTGAAAACTGCTGAGAGAA | TTGAGAAAACGAGTGCTTTGG |
| hsa_circ_0104689 | CCAGCATTGCCTCTGATACG | GAAGTCAGGGAAGTTTCTGCC |
| β-actin | CACCTTCTACAATGAGCTGCGTGTG | ATAGCACAGCCTGGATAGCAACGTAC |

**Supplementary Table 3** The list of primers of miRNAs for RT-qPCR

| Names of miRNAs | Reverse Transcription Primers 5’-3’ | Forward Primers 5’-3’ | Reverse Primers 5’-3’ |
| --- | --- | --- | --- |
| hsa-miR-135b-5p | GTCGTATCCAGTGCGTGTCGTGGAGTCGGCAATTGCACTGGATACGACTCACAT | GGTATGGCTTTTCATTCCT | CAGTGCGTGTCGTGGAGT |
| U6 | AACGATTCACGAATTTGCGT | CTCGCTTCGGCAGCACATA | AACGATTCACGAATTTGCGT |

**Supplementary Table 4** Clinicalpathology of 20 breast cancer patients(Cohort 2)

| Clinicalpathology of 20 breast cancer patients | | |
| --- | --- | --- |
|  | N | % |
| Age |  |  |
| ＞50 | 12 | 60 |
| ≤50 | 8 | 40 |
| pT |  |  |
| 1(≤2cm) | 12 | 60 |
| 2(＞2cm,≤5cm) | 8 | 40 |
| 3(＞5cm) | 0 | 0 |
| pN |  |  |
| 0 | 15 | 75 |
| 1(≥1,≤3) | 5 | 25 |
| 2(≥4,≤9) | 0 | 0 |
| 3(≥10) | 0 | 0 |
| ER |  |  |
| negative | 4 | 20 |
| positive | 16 | 80 |
| PR |  |  |
| negative | 5 | 25 |
| positive | 15 | 75 |
| HER2 |  |  |
| negative | 13 | 65 |
| positive | 7 | 35 |
| Ki67 |  |  |
| ≤20% | 5 | 25 |
| ＞20% | 15 | 75 |

**Supplementary Table 5** Clinicalpathology of breast cancer tissue microarrays (Cohort 3)

| Clinicalpathology of patients in breast cancer tissue microarrays | | |
| --- | --- | --- |
|  | N | % |
| Age |  |  |
| ＞50 | 54 | 63.53 |
| ≤50 | 30 | 35.29 |
| pT |  |  |
| 1(≤2cm) | 29 | 34.12 |
| 2(＞2cm,≤5cm) | 54 | 63.53 |
| 3(＞5cm) | 1 | 0.01 |
| pN |  |  |
| 0 | 45 | 52.94 |
| 1(≥1,≤3) | 18 | 21.18 |
| 2(≥4,≤9) | 12 | 14.12 |
| 3(≥10) | 7 | 0.08 |
| ER |  |  |
| negative | 20 | 23.53 |
| positive | 65 | 76.47 |
| PR |  |  |
| negative | 27 | 31.76 |
| positive | 58 | 68.24 |
| HER2 |  |  |
| negative | 62 | 72.94 |
| positive | 20 | 23.53 |
| Ki67 |  |  |
| ≤20% | 21 | 24.71 |
| ＞20% | 64 | 75.29 |
